# Ultrasound image dataset for ovarian follicular development detection in pigs

**DOI:** 10.1016/j.dib.2024.111033

**Published:** 2024-10-16

**Authors:** Zhicong Wang, Kexiong Liu, Yuqing Song, Qin Li, Lei An, Yan Liu, Jianhui Tian, Jiahua Bai, Shumin Wang

**Affiliations:** a Frontiers Science Center for Molecular Design Breeding (MOE), Key Laboratory of Animal Genetics, Breeding and Reproduction of the Ministry of Agriculture and Rural Affairs, State Key Laboratory of Animal Biotech Breeding, National Engineering Laboratory for Animal Breeding, College of Animal Science and Technology, China Agricultural University, Beijing 100193, China; b Institute of Animal Husbandry and Veterinary Medicine, Beijing Academy of Agriculture and Forestry Sciences, Beijing 100097, China

**Keywords:** Ultrasound, Pigs, Follicles, Animal reproduction

## Abstract

Pigs are one of the largest scale livestock species globally. In large-scale pig farms, the reproductive management of gilts and sows is a critical control point to ensure production capacity and reduce costs. More precisely, the understanding the developmental status of ovarian follicles in pigs is a critical basis for developing and improving management programme of oestrus detection and artificial insemination, a core component that determines sow production performance, but this knowledge is largely lacking. Ultrasound scan is a non-invasive and convenient method to understand the follicular developmental statues. However, there is currently a lack of reliable data to establish imaging standards, making it difficult to provide the most accurate support and reference for reproductive management in pigs. This dataset contains 868 images of ovarian follicles at different developmental stages in gilts and sows in favour of the identification and understanding of sow reproductive condition. To our knowledge, it is the inaugural dataset containing ultrasound images of large animals.

Specifications Table

**Table:** 

Subject	Animal science
Specific subject area	Pig reproduction: follicular development
Type of data	Ultrasound Images
Data collection	The high-quality ultrasound images were acquired using the Honda HS-1600V device (Honda Electronics, Tokyo, Japan). Initially, the images were gathered and categorized based on different treatments in pig farms. Subsequently, all images underwent thorough review and verification by the veterinarians from China Agricultural University and Institute of Animal Husbandry and Veterinary Medicine, Beijing Academy of Agriculture and Forestry Sciences.
Data source location	The ultrasound images was collected from four years from four different pig farms located in Beijing, China, the Great Wall Danyu livestock, Beijing Pig Breeding Co., Ltd. Pinggu branch, Nankou original breeding pig farm and Xishao original breeding pig farm.
Data accessibility	Repository name: Mendeley Data Data identification number: 10.17632/z6p55dvbxj.1 Direct URL to data: https://data.mendeley.com/datasets/z6p55dvbxj/1
Related research article	Liu K, Xu X, Song Y, Xiao L, Wen J, Ding H, Zhao S, Qiao D, Zhang B, Niu A, Bai J, Liu Y. Effect of altrenogest treatment before weaning on reproductive performance and production efficiency in primiparous and multiparous sows. Porcine Health Manag. 2024; 10:25. https://doi.org/10.1186/s40813-024-00377-7.

## 1 Value of the Data

• Ovarian ultrasound in livestock offers benefits such as being non-invasive, high accuracy in diagnosis, and easy operation. It is capable of quickly evaluating the development status of female ovarian follicles and help determine the estrus phase of females, which is crucial for accurately determining the timing of mating under large-scale breeding conditions, improving reproductive management efficiency and pregnancy outcomes, especially for intensive reproductive management of gilts and sows.
• This dataset is highly comprehensive, encompassing ultrasound images from gilts and sows, both of which are critical basic populations in pig breeding management.
• The dataset provides valuable resources for creating and developing machine learning to determine the ovarian status of gilts and sows, which will be beneficial for achieving large-scale rapid assessment of follicular development.
• This dataset provides valuable physiological data for researchers to gain a deeper understanding of reproductive regulation mechanisms of sows and gilts, which has been widely considered as a unique model of multiple-ovulating large animals.

## 2 Background

Pigs are one of the largest scale livestock species globally and a prominent source of meat, providing essential protein for the global food supply. Among the animals that are intensively farmed, pigs have been domesticated by humans with great success. The reproductive management programme of sows and gilts is crucial in large-scale intensive farming, serving as a critical control point to ensure production capacity and reduce costs. Both establishment and improvement of the sow breeding program largely depends on the accurate assessment of the developmental status of ovarian follicles and ovulatory dynamics in sows [1,2].

The B-ultrasound monitoring is a convenient, fast, and non-invasive method for examination and diagnosis, and has been widely used in the physiological and pathological detection and evaluation of human clinical conditions, including breast cancer, gallbladder diseases, fetal head biometry and model anima, etc., providing reference and basis for early diagnosis and treatment planning [[3], [4], [5], [6]]. In the field of animal husbandry, B-ultrasound monitoring has been practically applied in early pregnancy detection, which is essential for timely adjusting the management programme of pregnant and non-pregnant animals, thereby maximizing cost savings and increasing benefits [7,8]. However, accurate and reliable b-ultrasound monitoring needs a systematic and comprehensive basic data to establish imaging standards for the sow ovary monitoring. However, up to now, a large amount of reliable data of ovarian follicular ultrasound image remains lacking, either for natural estrus cycle or exogenous hormone-controlled estrus cycle, making it difficult to provide accurate reference for reproductive management programmes. Therefore, this paper presents a set of ovarian ultrasound image dataset for researchers in the field of large animal's reproductive physiology and pig breeders to use.

## 3 Data Description

Data was gathered from four pig farms, comprising more than 868 high-quality images of pig follicles. These images were compiled and formatted to support their application in machine learning and deep learning studies. The dataset is arranged in a standardized structure, showcasing various developmental stages of pig follicles for comparative evaluation. The images are systematically classified, facilitating easy navigation and access to images representing different developmental stages within the subfolders. This organized arrangement improves the dataset's usability for thorough research and analysis. In this investigation, we concentrate on identifying and analyzing four categories of developmental follicles: Small, Medium, Large, and Preovulatory [9].

### 3.1 Small follicles

**Fig. 1:**
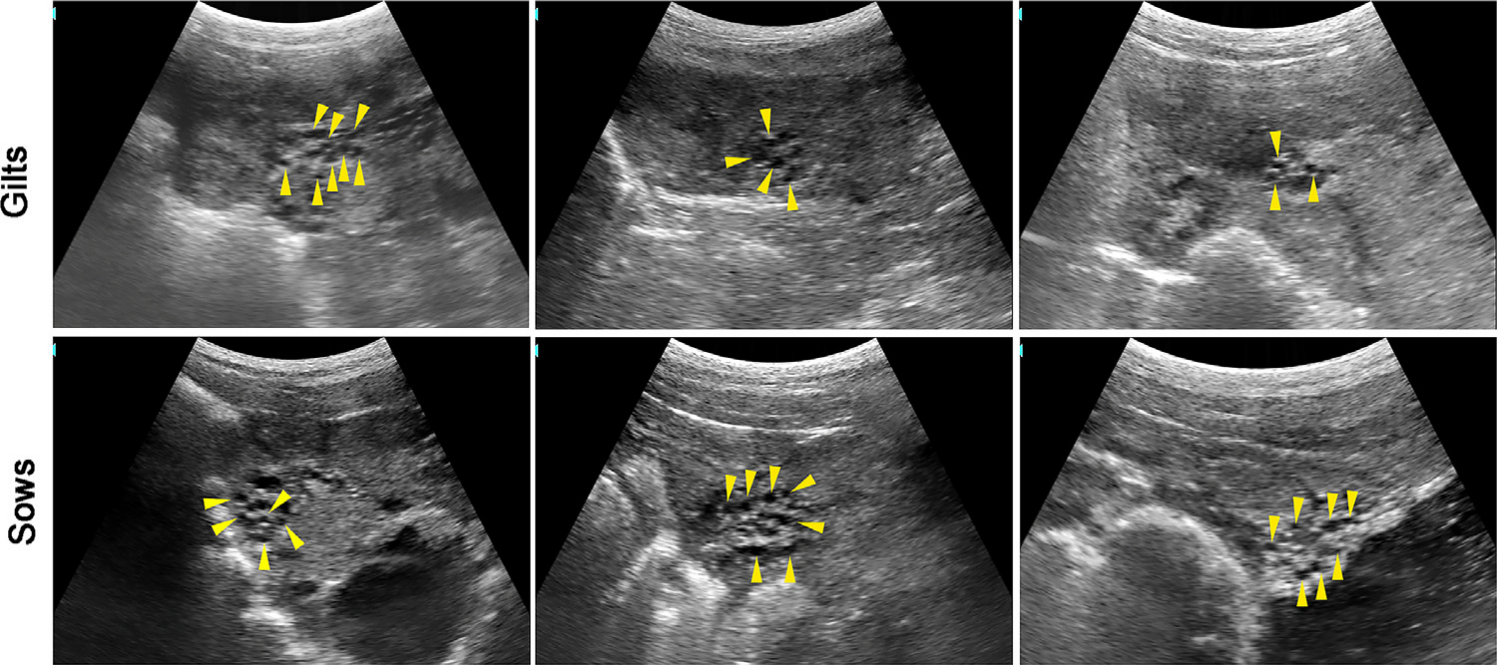
The images of small follicles in gilts and sows.

Fig. 1 illustrates the small follicles (<3 mm) in gilts and sows. During the luteal phase, sows in anestrus, progesterone inhibits the secretion of Gonadotropin-releasing hormone (GnRH) by the hypothalamus, thereby suppressing the pituitary secretion of gonadotropins and preventing follicle growth. At this period, the follicle size is typically relatively small, below 3 mm.

### 3.2 Medium follicles

**Fig. 2:**
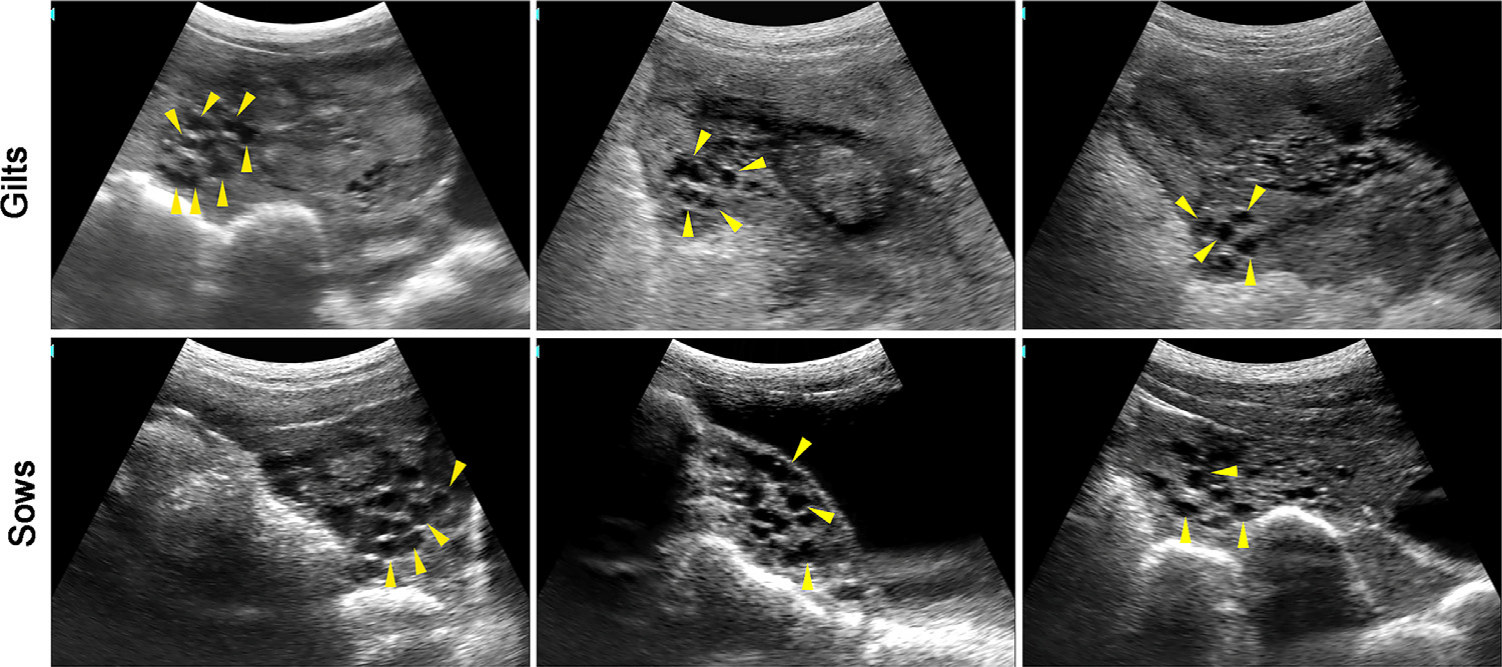
The images of medium follicles in gilts and sows.

Fig. 2 shows the medium follicle (3–5 mm) in gilts and sows. After luteolysis, the inhibition of GnRH secretion is removed and promoting the secretion of gonadotropins including follicle stimulating hormone (FSH) and luteinizing hormone (LH). During this period, the follicle size increase is mainly dependent on the action of FSH.

### 3.3 Large follicles

**Fig. 3:**
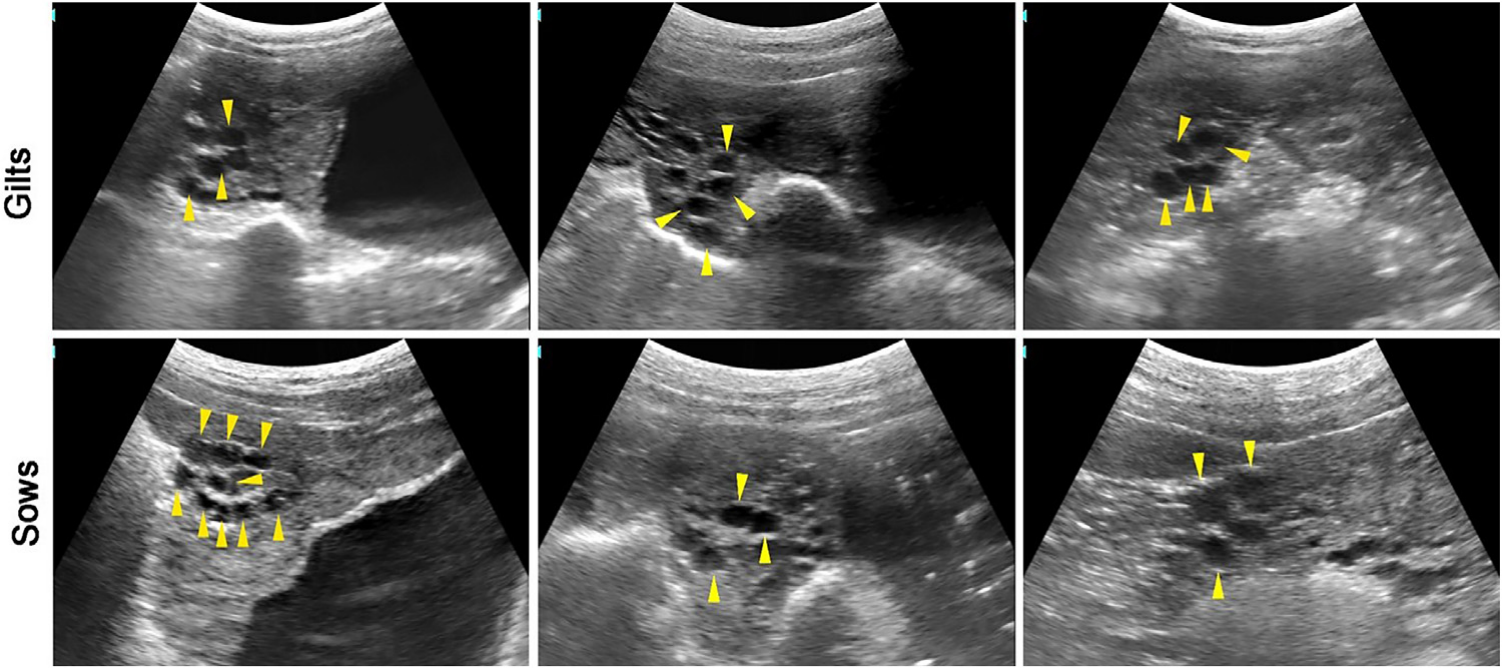
The images of large follicles in gilts and sows.

Fig. 3 displays the large follicle (5–6.5 mm) in gilts and sows. As the follicles develop, the ovaries will choose the dominant follicles while the smaller ones will undergo atresia. The dominant follicles will continue to grow until estrogen levels in the sow reach a certain threshold, promoting sow oestrus.

### 3.4 Preovulatory follicles

**Fig. 4:**
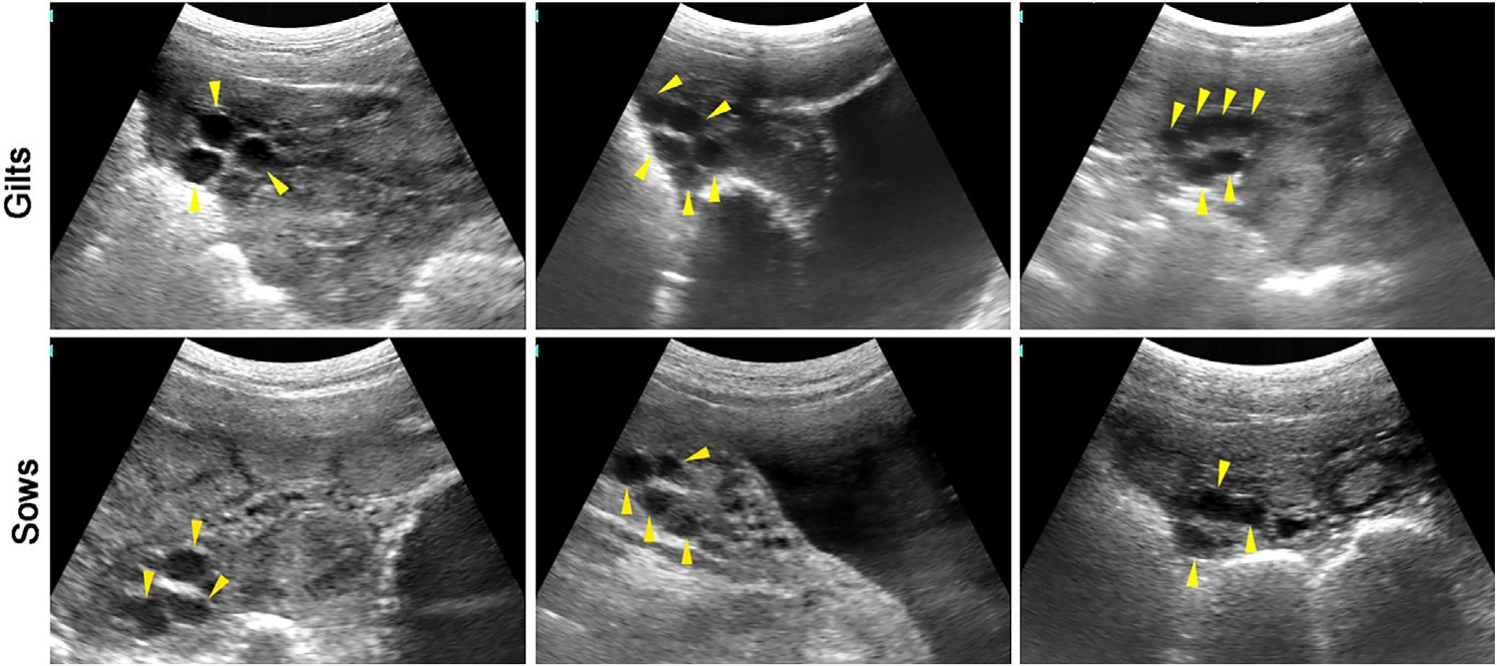
The images of preovulatory follicles in gilts and sows.

Fig. 4 illustrates the preovulatory follicles (>6.5 mm) in gilts and sows. After the onset of estrus, sustained estrogen stimulation will induce the hypothalamus to release GnRH, subsequently triggering the pituitary to secrete a significant amount of LH and form an LH surge. The LH surge further promotes follicle enlargement until ovulation occurs.

## 4 Experimental Design, Materials and Methods

### 4.1 Data collection

The dataset is composed of ultrasound images of the pig ovarian follicles. All these high-quality ultrasound images are collected by Honda HS-1600V device (Honda Electronics, Tokyo, Japan). The bulk of the training data was collected over the course of four years from four different pig farms located in Beijing, China, which are the Great Wall Danyu livestock, Beijing Pig Breeding Co., Ltd. Pinggu branch, Nankou original breeding pig farm and Xishao original breeding pig farm.

### 4.2 Collection of ultrasound images

Before using the device, ensure that it is complete and that the indicator is functioning properly. Verify the normal operation of the B-ultrasound machine. When testing follicles, locate the ovaries and ensure that the sows are standing and settled. Apply coupling agent to the probe of the B-ultrasound device, covering its head appropriately. Tilt the hairless area between 1 and 2 nipples in the lower abdomen of the sow to a 45-degree angle, then slowly move the probe close to the skin for a fan scan in various positions while minimizing damage and irritation during exploration. Once a clear image appears on screen, press freeze to analyze and measure follicle diameter [10].

### 4.3 Data process

To ensure the usefulness of the dataset, several tasks were carried out. This included the removal of duplicate or blurry images, resulting in a reduction of the number of ultrasound images to 868. The images were categorized into four files: Small follicle, Medium follicle, Large follicle, and Preovulatory follicle. Each files contains two subfiles: Gilt and Sow. We initially calibrated the image's brightness to enhance visibility. The size of the follicles was then measured using standard methods. Subsequently, all images were cropped to standard sizes using photoshop to remove unnecessary boundaries. The position of the follicle is indicated by the yellow triangle. The title of the image is composed of the pig type and its sequence within the subfiles. All images were checked by the veterinarians from China Agricultural University and Institute of Animal Husbandry and Veterinary Medicine, Beijing Academy of Agriculture and Forestry Sciences.

### 4.4 Data record

The ultrasound image dataset consists of 868 images, annotated, and verified by experienced veterinarian. It includes four classes according to follicular size. In total, 500 gilts and sows were involved in the data collection; the number of gilt images was 475, while the number of sow images was 393. The dataset consists of images with a resolution of 670×460 pixels and they are sorted into separate four folders named according to the content. Table 1 shows the number of images contained in each folder.

**Table 1:** Dataset sample distribution.

Follicular size (folder name)	Pig type (subfolder name)	Number of images
Small follicle	Gilt	61
	Sow	119
Medium follicle	Gilt	146
	Sow	132
Large follicle	Gilt	207
	Sow	103
Preovulatory follicle	Gilt	61
	Sow	39

## Limitations

The dataset has several limitations, including a lack of ultrasound images depicting pathological states of sow ovaries, such as follicular cysts. Attention should be given to increasing the collection of these images in subsequent data gathering. Additionally, due to sow movement during monitoring, ultrasound image quality may not always meet satisfactory results. Therefore, it is recommended that monitoring be conducted when sows are calm, such as during feeding times, to obtain clearer images.

## Ethics Statement

The animal procedures used in the experiments strictly adhere to the Beijing Municipal Laboratory Animal Welfare and Ethics Guidelines and are conducted in accordance with the regulations established by the China Agricultural University Animal Welfare and Animal Experiment Ethics Committee. All experimental animal usage is approved by the China Agricultural University Animal Welfare and Ethics Committee (AW80804202-1-1).

## CRediT Author Statement

**Zhicong Wang, Kexiong Liu:** Conceptualization, Methodology, Software. **Jiahua Bai**: Data curation. **Kexiong Liu:** Writing, Original draft preparation. **Yuqing Song**: Visualization, Investigation. **Shumin Wang** and **Qin Li:** Supervision. **Lei An:** Writing- Reviewing and Editing. **Yan Liu** and **Jianhui Tian**: Funding acquisition, Project administration

## Data Availability

Mendeley DataUltrasound image dataset for ovarian follicular development detection in pigs (Original data)
